# Postoperative Rehabilitation May Reduce the Risk of Readmission After Groin Hernia Repair

**DOI:** 10.1038/s41598-018-25276-0

**Published:** 2018-04-30

**Authors:** Grégoire Mercier, Jessica Spence, Christelle Ferreira, Jean-Marc Delay, Charles Meunier, Bertrand Millat, Tri-Long Nguyen, Fabienne Seguret

**Affiliations:** 1 Economic Evaluation Unit, Montpellier University Hospital, Montpellier, France; 2 Departments of Anaesthesia and Clinical Epidemiology and Biostatistics, McMaster University, Ontario, Canada; 3 Unit of Evaluation and Epidemiologic Studies on National Hospitalization Activity Databases, Department of Epidemiology, Biostatistics and Medical Information, Montpellier University Hospital, Montpellier, France; 4 Department of Anaesthesia and Critical Care B (DAR B), Montpellier University Hospital, Montpellier, France; 5 Department of Digestive Surgery, Montpellier University Hospital, Montpellier, France; 6 Department of Epidemiology, Biostatistics and Medical Information, Montpellier University Hospital, Montpellier, France; 7 Laboratory of Biostatistics, Epidemiology, Clinical Research and Health Economics, EA2415, University Institute of Clinical Research, Montpellier University, Montpellier, France

## Abstract

Thirty-day readmission after surgery has been proposed as a quality-of-care indicator. We explored the effect of postoperative rehabilitation on readmission risk after groin hernia repair. We used the French National Discharge Database to identify all index hospitalizations for groin hernia repair in 2011. Readmissions within 30 days of discharge were clinically classified in terms of their relationship to the index stay. We used logistic regression to adjust the risk of readmission for patient, procedure and hospital factors. Among 122,952 index hospitalizations for inguinal hernia repair, 3,357 (2.7%) related 30-day readmissions were recorded. Reiterated analyses indicated that readmission risk was consistently associated with patient complexity: age (per year after 60 years, OR 1.03, 95% CI 1.02–1.03, P < 0.001), hospitalization within the previous year (OR 1.56, 95% CI 1.44–1.69, P < 0.001), and increasing severity and combination of co-morbidities. Postoperative rehabilitation was identified as a protective factor (OR 0.56, 95% CI 0.46–0.69, P < 0.001). Older patients and those with greater comorbidity are at elevated risk of readmission after inguinal hernia repair. Postoperative rehabilitation may reduce this risk. Further studies are warranted to confirm the protective effect of postoperative rehabilitation.

## Introduction

Inguinal and femoral (groin) hernia repairs are two of the most common general surgical interventions, with more than 770,000 procedures being performed each year in the United States (US)^1^ and 160,000 in France^2^. These operations are typically performed as outpatient or short-stay procedures, with most admissions lasting no more than 1–2 days^3,4^. However, as many as 5–6% of patients may be readmitted in the 30 days after discharge from hospital^4,5^. Because of the associated health and financial implications, 30-day readmission has been proposed as a quality of care indicator^6^. Since the implementation of the Hospital Readmissions Reduction Program in the US^7^, numerous studies focusing on predicting and preventing thirty-day readmission have been published.

Given that age and number of comorbidities^8–11^ have been repeatedly identified as predictors and that studies evaluating the impact of changes to surgical^12–16^ and anaesthetic^14,17^ technique have been inconclusive, several researchers have questioned whether readmission is even preventable^18^. Given this possibility, there has been a recent interest in the role of postoperative strategies to prevent readmission^11,19–27^, including inpatient rehabilitation, home healthcare, long-term hospital stays, and discharge to a skilled nursing facility^21^. On one hand, it has been suggested that maintenance of care after surgery might reduce risk of readmission^19,24,25^. On the other hand, discharge to a post-acute care facility was an independent predictor of readmission after surgery^22,23,26,27^. Inpatient rehabilitation constitutes one of the only active interventions, as opposed to simply providing prolonged institutional care. We sought to explore the effect of inpatient rehabilitation on readmission after primary groin hernia repair.

## Methods

We conducted a retrospective cohort study using the French National Discharge Database. This exhaustive database contains public and private hospital discharge records, including diagnosis, treatment and demographic information. Since its inception on January 1^st^, 2004, this database has incorporated almost 24 million new records annually with a current total of more than 190 million entries. All methods were carried out in accordance with relevant guidelines and regulations. The study protocol was approved by the French institutional committee National Commission for Information Technology and Civil Liberties (*Commission Nationale de l*’*Informatique et des Libertés*). According to French law, informed consent is not required for retrospective analyses of discharge databases.

We included all patients aged 18 years or older who underwent a primary groin hernia repair between January 1st and December 31st of 2011. The records associated with an inguinal/femoral hernia surgery code (French Common Classification of Medical Procedures, CCAM^28^, 23^th^ version) were identified. Second, we selected patients with a primary diagnosis of inguinal/femoral hernia-associated symptoms (i.e., vomiting, nausea, bloating and constipation) (International Classification of Diseases, 10^th^ revision^29^, ICD-10 codes K40–46, K55, K56 K63, K65, K66, and R10, R11). We included only records within groin hernia repair diagnosis-related groups (DRGs, version 12. 11b)^30^. Finally, because hernia recurrence has been described to increase the risk of adverse postoperative outcomes^14,31^, we restricted our selection to cases defined as incidents by excluding patients with previous diagnoses of groin hernia between 2004 and 2011. We used anonymized patient identifiers to process the data and to eliminate record duplications.

We defined the primary outcome as the occurrence of 30-day readmission related to the index stay. As the worst possible outcome, we also included death prior to initial hospital discharge in our primary endpoint. To evaluate whether readmission was related to index stay, a digestive surgeon (hernia surgery is performed by digestive surgeons in France) and an anaesthesiologist independently reviewed all 30-day readmissions based on a method similar to a previously described one^32^. Considering the discharge-to-readmission time, age and principal diagnosis, readmissions were classified into 4 groups: “probably related to the primary surgery (local complication)”, “probably related to the primary surgery (general complication)”, “potentially related to the primary surgery”, or “probably not related to the primary surgery.” This last group was excluded from the primary analysis. Inter-rater agreement was evaluated using Cohen’s kappa coefficient.

To evaluate the effect of postoperative rehabilitation on readmission to the hospital, we adjusted for several patient-, procedure-, and hospital-related variables. Since a spline regression suggested that age had virtually no effect on the readmission risk below 60, we included age as a continuous variable above 60 years. Because we hypothesized that readmission risk could be explained by the combination of multiple pathologies, we created a discrete co-morbidity variable. In doing so, we first conducted a data mining algorithm to generate association rules^33^, which allowed us to identify certain combinations of diseases^34^ associated with readmission. Co-morbidities were included in the model according to their diagnostic pool, as defined in the ICD-10. Severity was categorized in five groups according to the DRG-grouping function^30^: from J (same-day surgery) to 1, 2, 3 and 4 (inpatient surgery of increasing risk). The surgical technique (laparoscopic or open repair; use of mesh or no), anatomic localization of the hernia (femoral or inguinal; unilateral or bilateral), diagnosis of hernia complications (obstruction and infection), hospitalization during the previous year, concomitant surgery, emergency admission and patient transfer were introduced as binary variables. The length of stay, driving time to hospital, hospital surgical volume (percentage of surgical stays among all hospital stays per year) and hospital activity range (number of different DRGs per year) were categorized into quartiles. The total number of stays per year for each hospital was logarithmically transformed and introduced as a continuous variable.

We compared the medians of continuous variables using the Wilcoxon-Mann-Whitney test and proportions using the *χ2* test for all categorical variables. Variables with p < 0.25 in univariate analyses were selected for inclusion in our multivariate model using stepwise selection (criterion alpha = 0.05). Variables meeting our criterion alpha were included in the final multivariate logistic regression model. The association of covariates with readmission after surgery was described using the odds ratios (OR) with a 95% confidence interval (CI). Adjusted effects with P < 0.05 were considered to be statistically significant.

Given that there is a risk of selecting spurious variables when model selection is used in large datasets, we evaluated the robustness of our results by reiterating stepwise selection on random subsamples of varying size^35,36^, with 100 iterations conducted at each step. The analyses were carried out with SAS Enterprise Guide 4.3 (SAS Institute Inc., Cary, NC, USA) and R 2.15.2 (R Development Core Team. R Foundation for Statistical Computing, Vienna, Austria).

### Data availability

The data that support the findings of this study are available from Fédération Hospitalière de France, but restrictions apply to the availability of these data, which were used under license for the current study, and so are not publicly available. However, the data are available from the authors upon reasonable request and with the permission of Fédération Hospitalière de France.

## Results

**Figure 1:**
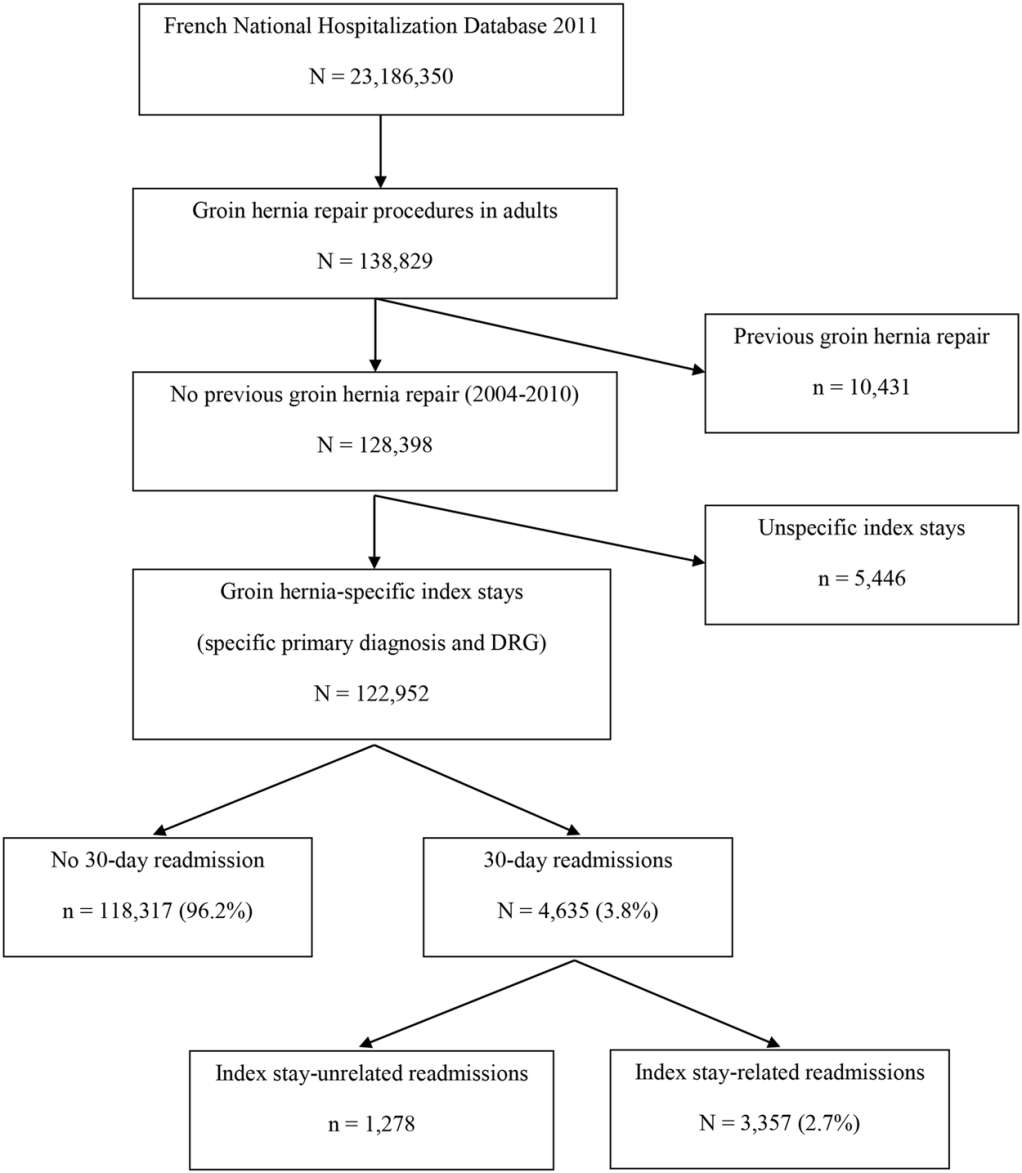
Admissions flow chart.

A total of 122,952 patients underwent a primary groin hernia repair (Fig. 1). The median age was 61 years, and 106,925 (87.0%) were males; 4,694 admissions (3.8%) were emergency hospitalizations, and 527 (0.5%) were transfers from care units. Most procedures were open techniques (81,288, 66.9%) and used mesh (110,117, 89.6%). Only 48,352 (39.3%) procedures were same-day surgeries.

In our database, 4,409 patients (3.6%) were re-hospitalized within 30 days after hernia surgery, and 226 (0.2%) died in the hospital. As shown in Fig. 1, 3,357/4,635 events were probably or potentially related to the index hospitalization (inter-rater Cohen’s kappa coefficient = 0.845, 95% CI 0.827–0.863). The median interval between discharge and readmission was 8 days. The most common reasons for readmission were post-procedural complications (haemorrhage/haematoma and digestive disorders) (7.0%), acute abdominal and pelvic pain (5.4%), and retention of urine and haematuria (4.9%) (Table 1).

**Table 1:** Description of the primary composite outcome of death or 30-day readmission related to the index stay (with top five most frequent reasons).

	n (%)	ICD-codes
***In*** **-** ***hospital deaths during the index stay***	**226** (**6.7**)	
***Readmissions probably related to the index stay*** (***complications in the operative field occurring within 7 days***)	**1**,**355** (**40.4**)	
Post procedural complications: haemorrhage/haematoma, digestive disorders	235 (7.0)	T81, K91
Acute abdominal and pelvic pain	181 (5.4)	R10
Constipation, obstruction and paralytic ileus	113 (3.4)	K56, K59
Retention of urine, haematuria	164 (4.9)	R31, R33
Cutaneous abscess, furuncle and carbuncle	92 (2.7)	L02
***Readmissions probably related to the index stay*** (***general complications occurring within 7 days***)	**512** (**15.2**)	
Symptoms, signs and abnormal clinical findings: ascites, respiratory disorders, disturbance of skin sensation, symptoms of systemic inflammation and infection, fever, malaise and fatigue, syncope and collapse, shock	137 (4.1)	R06, R07, R18, R20, R50, R53, R55, R57, R65
Phlebitis and thrombophlebitis, pulmonary embolism, arterial embolism and thrombosis, ischaemic stroke, ischaemic heart disease, atrial fibrillation and flutter, heart failure, acute kidney failure	114 (3.4)	I20, I21, I26, I48, I50, I63, I64, I74, I80, N17
Cystitis and other genitourinary disorders	80 (2.4)	N17, N30, N39, N40, N47
Pneumonia and other respiratory diseases	31 (0.9)	J15, J18, J44, J98
Gastric ulcer, acute appendicitis, and other digestive diseases	13 (0.4)	K25, K35, K92
***Readmissions possibly related to the index stay*** (***complications occurring after 7 days***)	**1**,**264** (**37.7**)	
Superficial injury of the abdomen, lower back, pelvis or external genitals	168 (5.0)	S30
Hypertension, ischaemic stroke, ischaemic heart disease, cardiomyopathy, cardiac dysrhythmia, heart failure	165 (4.9)	I10, I20, I21, I25, I42, I44, I47, I48, I50, I63, I70
Inguinal hernia, ventral hernia, gastritis and duodenitis, cholelithiasis, and other digestive diseases	147 (4.4)	K29, K40, K43, K80, K92
Cutaneous abscess, furuncle and carbuncle	107 (3.2)	L02
Return hospital visit for follow-up examination, aftercare adjustment and management	127 (3.8)	Z04, Z09, Z45, Z46, Z51, Z71
***Other readmissions*** (***not considered as outcomes***)	**1**,**278**	

Only 1,787 patients (1.4%) received postoperative rehabilitation. Of these, 9.2% were readmitted within 30 days, compared with 2.6% of those not discharged to a rehabilitation unit (unadjusted P < 0.001).

To explain the readmission risk, all covariates were introduced into a multivariate logistic regression, except the uni- or bi-laterality of the repair and driving time to hospital (P = 0.628 and P = 0.679 in univariate analysis). After adjustment, postoperative rehabilitation was significantly associated with readmission risk reduction, OR 0.56 (95% CI 0.46–0.69, P < 0.001).

Sixteen patient-related, institutional and admission factors were also significantly associated with readmission risk. Age greater than 60, increasing number of co-morbidities combinations, severity, hospitalization during the previous year, pre- or postoperative admission to an intensive care or step down unit, emergency admission, admission by transfer from another inpatient facility, preoperative complicated groin hernia (i.e., hernia strangulation, with or without infection), concomitant surgical procedure and discharge to other acute care facilities were all associated with increased odds of readmission. Female sex, mesh repair, laparoscopic technique and higher hospital surgery volume were all associated with lower odds of readmission. The odds ratios are reported in Table 2. The model yielded satisfactory fit measures (c-statistic: 0.725, Hosmer-Lemeshow test P = 0.488). All-cause readmission analysis (not reported) generated similar results but with lower discriminative performance (c-statistic: 0.698).

**Table 2:** Adjusted effects on readmission risk.

		n (%)	Odds ratio (95% CI)	*P*
Age	Year over 60	63,056 (51.3)	1.03 (1.02, 1.03)	<0.001
Sex	Female	16,027 (13.0)	0.89 (0.80, 0.98)	0.021
Co-morbidities*	Class 0	75,185 (61.1)	1.00	
	Class 1	25,652 (20.9)	1.17 (1.06, 1.29)	0.002
	Class 2	327 (0.3)	1.20 (0.65, 2.22)	0.565
	Class 3	12,064 (9.8)	1.38 (1.23, 1.55)	<0.001
	Class 4	5,514 (4.5)	1.62 (1.41, 1.87)	<0.001
	Class 5	2,615 (2.1)	2.51 (2.13, 2.97)	<0.001
	Class 6	398 (0.3)	3.51 (2.58, 4.76)	<0.001
	Class 7	691 (0.6)	3.06 (2.40, 3.89)	<0.001
	Class 8	506 (0.4)	4.32 (3.34, 5.59)	<0.001
1-year prior hospitalization	Yes	19,071 (15.5)	1.56 (1.44, 1.69)	<0.001
Complicated hernia diagnosis	Yes	5,936 (4.8)	1.20 (1.03, 1.39)	0.018
Severity level	Same-day surgery	48,346 (39.3)	0.78 (0.71, 0.86)	<0.001
	1 (low)	61,688 (50.2)	1.00	
	2	9,828 (8.0)	1.36 (1.21, 1.52)	<0.001
	3	2,465 (2.0)	1.48 (1.25, 1.75)	<0.001
	4 (high)	625 (0.5)	1.88 (1.46, 2.42)	<0.001
Emergency entrance	Yes	4,694 (3.8)	1.28 (1.10, 1.49)	0.002
Transfer at entrance	Yes	447 (0.4)	1.50 (1.11, 2.02)	0.008
Surgery technique	Laparoscopy	41,664 (33.9)	0.83 (0.76, 0.91)	<0.001
Mesh repair	Yes	110,117 (89.6)	0.88 (0.79, 0.98)	0.017
Concomitant procedure	Yes	30,873 (25.1)	1.28 (1.18, 1.40)	<0.001
Intensive care unit	≥1 day	181 (0.1)	2.60 (1.82, 3.70)	<0.001
Step down unit	≥1 day	560 (0.5)	1.51 (1.19, 1.91)	0.001
Transfer to acute care	Yes	592 (0.5)	3.95 (3.16, 4.94)	<0.001
Post-acute rehabilitation	Yes	1,787 (1.5)	0.56 (0.46, 0.69)	<0.001
Hospital surgery volume	1st quartile (≤25%)	30,092 (24.5)	1.00	
	2nd quartile (26–30%)	28,638 (23.3)	0.93 (0.85, 1.02)	0.128
	3rd quartile (31–45%)	32,116 (26.1)	0.81 (0.73, 0.90)	<0.001
	4th quartile (>45%)	32,106 (26.1)	0.79 (0.70, 0.90)	<0.001
Hospital activities diversity^†^	1st quartile (≤300 DRG/year)	32,408 (26.4)	1.00	
	2nd quartile (301–400)	44,596 (36.3)	1.05 (0.94, 1.18)	0.374
	3rd quartile (401–500)	28,817 (23.4)	1.10 (0.96, 1.25)	0.169
	4th quartile (>500)	17,131 (13.9)	1.25 (1.08, 1.44)	0.002

^†^DRG, diagnosis-related groups.

*Co-morbidity classes: (**0**) no condition; (**1**) other conditions; (**2**) infectious and parasitic diseases; (**3**) diseases of the digestive, respiratory, genitourinary, or nervous system, or injury, poisoning or certain other consequences of external causes; (**4**) any combinations between diseases of the digestive, respiratory, genitourinary, or circulatory system, or endocrine, nutritional or metabolic diseases, or congenital malformations, deformations or chromosomal abnormalities, or injury, poisoning or certain other consequences of external causes; (**5**) any combinations of symptoms, signs and abnormal clinical and laboratory findings, not elsewhere classified with any other diseases; (**6**) any combinations of symptoms, signs and abnormal clinical and laboratory findings not elsewhere classified with diseases of the genitourinary system; (**7**) any combinations of symptoms, signs and abnormal clinical and laboratory findings not elsewhere classified with diseases of the digestive system; (**8**) any combinations of symptoms, signs and abnormal clinical and laboratory findings not elsewhere classified with diseases of the respiratory system.

**Figure 2:**
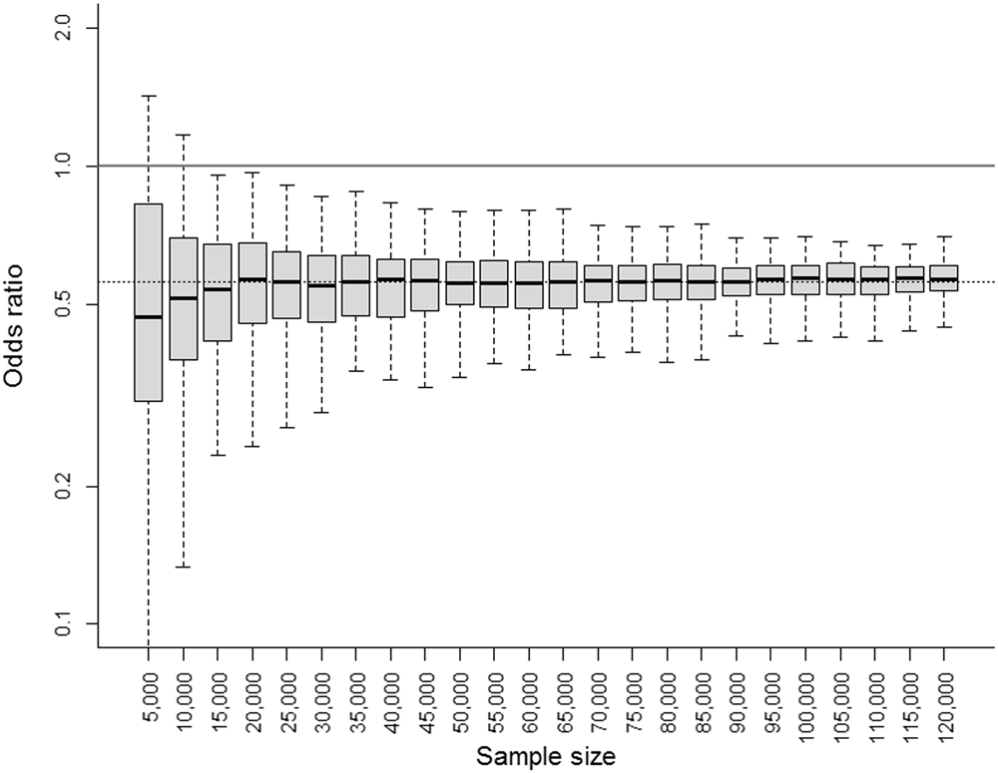
Sensitivity analysis for re-estimating the effect of rehabilitation on 30-day readmission in random subsamples of varying size.

We conducted a sensitivity analysis by reiterating model selection on random subsamples of varying size. The protective effect of post-acute rehabilitation was robust against random fluctuations and size variation (Fig. 2), with a consistent statistical significance (achieved in 100% of samples with N = 120,000). Factors related to patient complexity (age, previous hospitalization in the preceding 12 months, co-morbidities and severity) and laparoscopy also showed consistent effects on readmission (>80% of samples with N = 120,000).

## Discussion

Our study showed a low rate of 30-day readmission after groin hernia repair, which was lower than the rates reported in previous studies^4,5^. As expected, the readmission risk was mostly explained by patient-related factors^37^, and as in previous studies^8,10,11^, increasing age, co-morbidity and history of hospitalization in the last 12 months were associated with higher readmission risks. The 30-day readmission risk gradually increased in the presence of multiple co-morbidities, particularly when several common chronic diseases were associated (e.g., hypertension, diabetes, hyperlipidaemia, COPD). Also, emergency repair remained associated with higher risk of adverse postoperative outcomes, including both readmission and death^10,14,38^. In keeping with previous work, our study indicated that the use of mesh and laparoscopic techniques were protective factors^31^. In terms of structural variables, readmission risk was reduced when surgery occurred in hospitals with high surgery volumes, while the number of stays per year seemed to have no effect, similar to what has been previously reported in the literature for major surgery^23^. In our study, same-day surgery was relatively uncommon, which may be due to both differences in definition and a late implementation of this practice in France compared to North American and other European countries^39^. We paradoxically found a protective effect for readmission that may be explained by the use of same-day surgery being reserved for healthier patients^32^.

To prevent spurious associations related to the large size of our cohort, we repeated our analyses on random subsamples of varying size. As expected, factors related to patient complexity (i.e., age, hospitalization in the preceding 12 months, co-morbidities and severity) were consistently significant and related to both readmission and adverse perioperative outcome, including death. Given this finding, consideration should be given to re-evaluating the indications for elective hernia repair, particularly in elderly and multi-morbid patients. For an asymptomatic hernia, a wait-and-see policy may merit consideration^40^. In situations where emergency surgery is required, anticipating postoperative interventions to mitigate readmission may be beneficial. Further studies investigating the impact of personalized rehabilitation programmes on readmission for comorbid patients would be of high interest.

Recent studies have emphasized the importance of post-acute care after surgery^11,19–27^, which we found may prevent short-term readmission. Our findings contradict previous studies showing an elevated risk of readmission in patients discharged to poste-acute care facilities after surgery^22,23,26,27^. These studies evaluated discharge to any post-acute care facility, without accounting for whether they were institutional or rehabilitative placements. Nonetheless, given that the readmission rate after groin hernia repair is low, the absolute risk reduction attributable to postoperative rehabilitation may be limited. Further studies are needed to evaluate its effect, specifically in high-risk patients and to identify which patients will benefit the most from these transitional interventions^20^. Similarly, interventions such as pre-habilitation might reduce the readmission risk in digestive surgery^41,42^. Pre-habilitation was not implemented in France until 2011, and further studies on this specific topic might be useful.

Our study has several limitations. Several potential predictors of readmission were not available in our database, including American Society of Anaesthesiologists (ASA) class^9^, anaesthesia modality^14,17^, use of anticoagulant medication^43^ and functional and social variables^20^. Our adjustment may also have been limited by the absence of randomization, which would not consider selection bias (e.g., the protective role of laparoscopy), and by the underreporting of co-morbidities, which is a recognized shortcoming in our dataset. Given the non-randomized design, the protective effect of rehabilitation needs to be confirmed in either experimental or observational studies that use methods for reducing model dependence^44^. Since this study explores the effect of numerous variables on the outcome, we chose to perform a regression adjustment, instead of nonparametric matching methods, which allows the causal effect of one variable at a time to be estimated. Being therefore exposed to model-dependence and model-extrapolation issues, the protective effect of rehabilitation must be confirmed in further studies. As some variables related to both the outcome and the rehabilitation status were missing from our database (e.g., social variables), we did not perform a propensity score analysis. The rate of day-case surgery was lower in this study than it is now in most health systems. However, this does not threaten the overall validity of the results because the current evidence comparing same-day versus overnight stay surgery for digestive surgery (including hernia repair) suggests no difference in terms of readmission rate^45^. Finally, the 30-day threshold for readmission is debatable as an outcome of importance, given that adverse postoperative outcomes after hernia repair tend to occur beyond this time period^31^. Our qualitative evaluation of the link between readmission and the index surgery was important for reducing this potential bias^18,32,46^.

Despite these identified limitations, our study has several strengths. We obtained good inter-rater reliability in determining the relationship between the index procedure and the need for readmission. Second, we used a unique approach for considering the role of comorbidities, which hypothesized that readmission risk could not only be affected by the presence of certain conditions but also their combination. Although we were not able to validate them, we used comorbidity combinations based on association rules instead of the indices described by Charlson^47^ and Elixhauser^48^. Indeed, the latter were developed to predict mortality, and attribute less weight to digestive diseases, compared to cardiac and vascular diseases. In addition, recent studies suggest that using them for nonfatal outcomes is flawed^49,50^. Third, our model had satisfactory goodness of fit, in spite of the acknowledged limitations of using administrative datasets for the development of models^10^. Administrative datasets also have strengths, in that they constitute a valid and low-cost source of clinical information^51–53^. We used an extremely large and exhaustive national database, limiting selection bias. However, an issue related to using such a large database is that statistically significant results that have little or no clinical significance may be identified^23,36^. To mitigate this, we reiterated our analysis on random subsamples to clearly distinguish real from spurious associations. The finding that a small number of the seventeen independent factors that were identified in the larger sample remained constant warns against two issues: the statistical instability of stepwise selection^35^ and the subsequent clinical consideration of factors that are not truly relevant. Our original approach did not involve only the identification of readmission factors but also the assessment of their pertinence. To the best of our knowledge, this study is the first to highlight a protective effect of post-surgical rehabilitation on 30-day readmission after inguinal and femoral hernia repairs, the two most common surgical interventions. These findings, which may lead to improvements in postoperative care, need to be confirmed in future studies.

## Conclusions

Our study shows that readmission after groin hernia repair is largely explained by patient complexity but may be prevented by post-acute rehabilitation. Further study is required to confirm our results and to develop individual risk prediction tools that could allow clinicians to detect higher risk patients to whom such postoperative interventions should be applied.
